# An In Vitro Assessment of the Shear Bond Strength of Alkasite Restorative Material in Primary Molars Compared with Glass Ionomer and Resin-Modified Glass Ionomer Restorations

**DOI:** 10.3390/ma17246230

**Published:** 2024-12-20

**Authors:** Ayman M. Sulimany, Mannaa K. Aldowsari, Saad Bin Saleh, Sarah S. Alotaibi, Bushra M. Alhelal, Hebah M. Hamdan

**Affiliations:** 1 Department of Pediatric Dentistry and Orthodontics, College of Dentistry, King Saud University, Riyadh 11545, Saudi Arabia; maldowsari@ksu.edu.sa (M.K.A.); ssbinsaleh@ksu.edu.sa (S.B.S.); 2 College of Dentistry, King Saud University, Riyadh 11545, Saudi Arabia; saraotaibi99@gmail.com (S.S.A.); bushraalhelal@gmail.com (B.M.A.); 3 Department of Periodontics Dentistry and Community Dentistry, College of Dentistry, King Saud University, Riyadh 11545, Saudi Arabia; hhamdan1@ksu.edu.sa

**Keywords:** alkasite, GIC, primary molars, RMGIC, shear bond strength

## Abstract

(1) Background: Alkasite is a novel restorative material that has attracted interest in recent years because of its distinctive characteristics, including its high translucency and excellent biocompatibility. It is comparable to glass ionomer cement (GIC) and resin-modified glass ionomer cement (RMGIC) due to its fluoride-release ability and usage in esthetically concerned areas. This study aimed to assess the shear bond strength (SBS) of Alkasite restorative material in comparison with GIC and RMGIC (2) Methods: The study sample included 120 extracted sound primary molars and was randomly split into three groups, including group 1: RMGIC; group 2: Alkasite; and group 3: GIC. Each group was then sub-grouped into immediate and delayed loading. SBS was measured for each group using a universal testing machine. One-way analysis of variance with Tukey’s post hoc test and an independent t-test were used for statistical analyses (3) Results: The immediate SBS was higher in Alkasite, followed by RMGIC and GIC, with means of 10.84 ± 1.96, 10.64 ± 1.74, and 6.09 ± 1.75, respectively. However, there was no significant difference between Alkasite and RMGIC (*p* = 0.94), whereas RMGIC and Alkasite showed significantly higher values than GIC, with *p* < 0.0001. Regarding delayed SBS, no significant difference was noted between Alkasite and RMGIC (*p* = 0.14), whereas both showed significantly higher values than GIC, with mean values of 6.30 ± 1.44, 5.556 ± 1.38, and 3.29 ± 0.61, respectively (*p* < 0.0001). (4) Conclusions: Our findings show that RMGIC and Alkasite have comparable outcomes to each other and better outcomes when compared with conventional GIC.

## 1. Introduction

Dental materials have been developed rapidly, starting with amalgam, which has classically been the only restoration material used in posterior teeth. Significant improvements were mainly applied to esthetic, mechanical properties, and wear resistance by introducing resin material that can preserve the natural tooth structure by creating a strong chemical bond between enamel and dentin [1]. Conventional glass ionomer cement (GIC), resin-modified GIC (RMGIC), and composite resins are the current predominant restorative materials used in pediatric dentistry [2].

Conventional GICs are acid–base reaction materials obtained from a powder–liquid mixture [3]. GIC has the advantages of having the same coefficient thermal expansion as natural tooth tissue, biocompatible material, chemical adhesion to teeth, low shrinkage, and minimal marginal leakage, and it induces remineralization in adjacent proximal caries [4]. RMGICs are similar to GICs combined with a radical polymerization reaction of methacrylate monomers. The main reason for adding resin is to improve mechanical performance and reduce the material’s sensitivity to early water/saliva contamination relative to GIC [3]. The ability of GIC and RMGIC to release fluoride helps in reducing the likelihood of secondary caries development [2]. Although the application of conventional GIC is confined to areas with low to moderate stress-bearing areas, it has notably shorter longevity when used in primary teeth, whereas RMGIC has improved retention ability, even with the polymerization shrinkage and volumetric changes in the material [2].

Alkasite or Cention N is a new restorative material that was introduced as a type of resin restorative material. It consists of an alkaline filler that can discharge fluoride ions. It can be cured chemically and has the option of being cured by light [2]. Alkasite also offers aesthetically pleasing tooth-colored properties along with excellent flexural strength [2]. The presence of an alkaline filler in this material triggers the release of hydroxide ions, which assist in maintaining a balanced pH level when exposed to acidic substances [5]. Consequently, this pH balance helps in preventing demineralization and allows for the substantial release of calcium and fluoride, aiding in the remineralization process of dental enamel. Alkasite offers the advantage of being easy to use [5] and can be used as a permanent restoration for different classes, such as Class I, II, and V [6]. Unlike traditional glass ionomer cement (GIC), it eliminates the need for a primer, curing device, and varnish as etching with phosphoric acid is not required, and the use of an adhesive is optional [6]. However, modifications to cavity preparation are essential if adhesives are not applied, as this provides an undercut for the retention of the restoration, similar to amalgam cavity preparation. If an adhesive is used, a minimally invasive cavity preparation concept is applied. Cention N consists of powder and liquid. Unlike composite materials, this material does not contain TEGDMA, Bis-GMA, or HEMA [7]. The compositions of the powder and liquid of Cention N are shown in Table 1.

Unlike traditional glass ionomer cement (GIC), it eliminates the need for a primer, curing device, and varnish. This streamlined approach reduces the number of work steps required, saving valuable time by eliminating up to seven additional phases in restoring cavities [5]. According to the manufacturer’s description, Alkasite exhibits an enhanced degree of polymerization, particularly in increment thicknesses exceeding 4 mm. This is made possible by its versatile nature as it can be used in both self-cured and dual-cured modes. By offering these options, the material ensures optimal polymerization, even in thicker increments, providing reliable performance in various clinical situations [12].

When dealing with pediatric patients, minimizing chair time and reducing the number of clinical steps is crucial due to the challenges posed by children’s limited ability to cooperate with the dentist. One approach to achieve this is by utilizing a surface treatment, which eliminates the need for etching, rinsing, and drying. By eliminating the need for any kind of treatment process, the overall time spent in the dental chair can be significantly reduced. This streamlined approach not only improves efficiency, but also helps to enhance the overall experience for young patients by minimizing the number of procedural steps they need to undergo [13].

Many studies have been conducted to measure different properties of Alkasite restorative material. Arjun et al. [14] and Priytama et al. [15] concluded that Alkasite has the least microleakage at the restoration interface compared to other restorative materials. Moreover, Alkasite was observed to have the highest fluoride ion release compared with GIC, as mentioned by Nupur et al. [16], and it was better than both RMGIC and GIC over time, as observed by Singh et al. [17].

Despite the increasing interest in Alkasite as a restorative material, there is a noticeable paucity of studies examining its shear bond strength (SBS), especially in primary teeth. Therefore, this study aimed to resolve this gap by evaluating the shear bond strength of Alkasite restorative material in comparison with glass ionomer and resin-modified glass ionomer restorative materials.

## 2. Materials and Methods

### 2.1. Study Design

**Figure 1:**
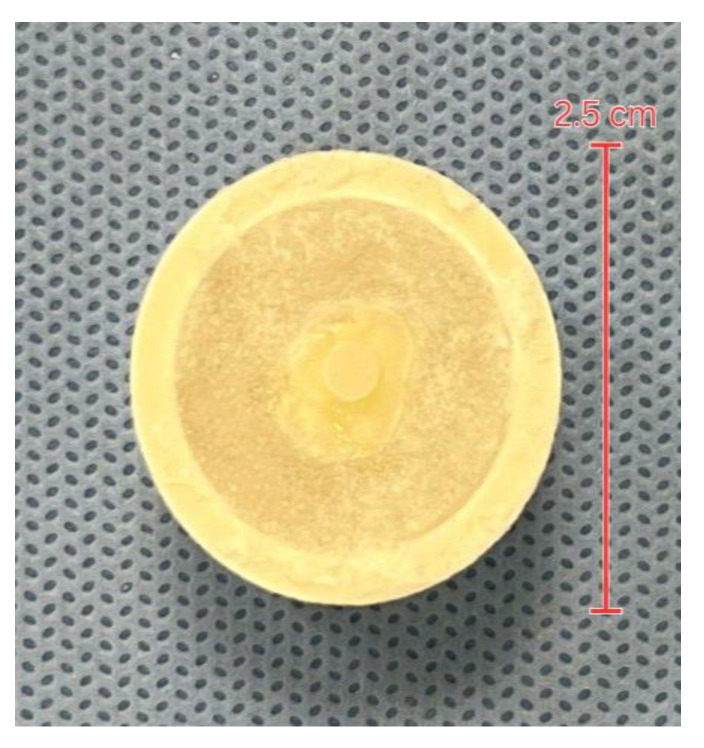
Specimens after tested material application.

One hundred and twenty human non-carious primary molars were collected from multiple dental clinics located in Riyadh, Saudi Arabia. The teeth were sterilized and stored in distilled water at room temperature until the time of the experiment. The radicular area of each primary molar was trimmed and embedded in plasticized polyvinyl chloride (PVC) of 2 cm length. The teeth were randomly divided into three groups, namely group 1: RMGIC; group 2: Alkasite; and group 3: GIC. Occlusal surfaces were trimmed to expose a flat occlusal surface of mid-coronal dentine. All specimens were stored in an incubator (Memmert Universal Oven, Germany) at 37.5 °C and 100% humidity until the day of the experiment. All specimens were rinsed and dried directly before the test material was applied. An impression was made on a columnar metal blank of 3 mm diameter and 2 mm height that was created using putty impression material to standardize the diameter of all testing materials in all samples. The putty rubber mold was carefully filled with test materials to prevent any air bubbles and voids. Then, the materials were placed on the flat dentin surface of specimens for all groups. The surface of the test material made complete contact with the flat dentin surface and avoided contact with the enamel surface (Figure 1). SDI Riva self-cure, SDI Riva light cure, and Alkasite were handled according to the manufacturer’s instructions (Table 1). Each group was then divided into immediate and delayed subgroups. The immediate subgroups were tested after 24 h of material application, whereas the delayed subgroups underwent artificial aging using a thermocycling machine (Huber, SD-Mechatronik-Thermocycler, Berching, Germany). The thermocycling process involved subjecting the samples to 5000 cycles at temperatures between 5 °C and 55 °C to simulate 6 months of physiological use.

### 2.2. Sample Size Calculation

The sample size was calculated based on the existing literature, taking into account a 95% confidence interval and 80% power for this study. To reach a significance level (α) of 0.05, an effect size of 0.45, and a power of 0.95, a minimum sample size of 120 was suggested.

### 2.3. SBS Test

**Figure 2:**
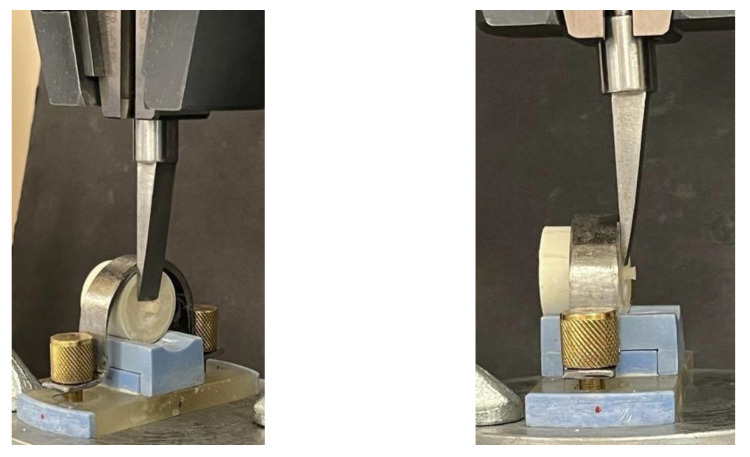
SBS testing at the interface between the tooth and tested material.

The bond strength was evaluated using a universal testing machine (Instron 5965, Norwood, MA, USA). The specimens were secured to the device using a metal mold. The materials were positioned at a right-angle to the dentin, and the plunger of the device was in contact with the specimens (Figure 2). The specimens were fixed to the table, and the device was operated at a controlled speed of 1 mm/min toward the plunger. The detachment of the tested materials from the dentin surface was calculated using specialized software (Bluehill 3).

### 2.4. Mode of Failure

To determine the failure pattern of the materials after SBS testing, the surfaces of the specimens were examined using a digital microscope (HIROX, KH-7700, digital microscope system, Tokyo, Japan).

### 2.5. Statistical Analysis

The Statistical Package for the Social Sciences (SPSS) (v.22.0, IBM, Chicago, IL, USA) was used for the statistical analysis. The mean and standard deviation values of the shear bond strength for all tested groups were compared using analysis of variance (ANOVA) with Tukey’s post hoc test. The differences in the immediate and delayed shear bond strength of each material were evaluated with an independent *t*-test. Fisher’s exact test was conducted to analyze the pattern of different failure modes.

## 3. Results

### 3.1. Shear Bond Strength

The SBS of the immediate groups is shown in Table 2. There was a statistically significant difference in the mean SBS between the study groups (*p* < 0.0001). The between-group analysis showed that there was a significant difference between the mean SBS values of the RMGIC and Alkasite groups compared with the GIC group (*p* = 0.0001) (Table 3). The mean SBS values of the RMGIC and Alkasite groups were significantly higher than that of the GIC group (10.64 ± 1.74, 10.84 ± 1.96, and 6.09 ± 1.75, respectively). However, no statistically significant differences were found between the mean SBS values of the RMGIC group and Alkasite group (*p* = 0.94).

The mean SBS of the delayed groups is shown in Table 4. There was a significant difference between the mean SBS among the study groups (*p* = 0.0001). The post hoc analysis showed that there was a significant difference between the mean SBS values of the RMGIC and Alkasite groups compared with the GIC group (*p* = 0.0001) (Table 5). The mean SBS values of the RMGIC and Alkasite groups were significantly higher than that of the GIC group (5.556 ± 1.38, 6.30 ± 1.44, and 3.29 ± 0.61, respectively). However, no significant difference between the mean SBS values of the RMGIC group and Alkasite group was found (*p* = 0.14).

Table 6 illustrates the differences between the immediate and delayed SBS values in each study material. The immediate SBS values were always higher than the delayed SBS values in all groups. However, there were no statistically significant differences in the mean SBS between the immediate and delayed subgroups in the RMGIC, Alkasite, and GIC groups (*p* = 0.91, 0.46, and 0.61, respectively).

### 3.2. Mode of Failure

**Figure 3:**
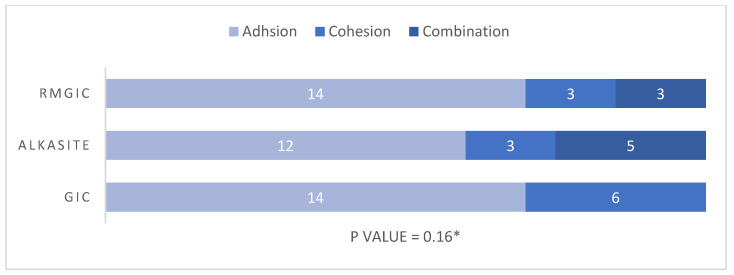
Immediate mode of failure. * Obtained using Fisher’s exact test.

**Figure 4:**
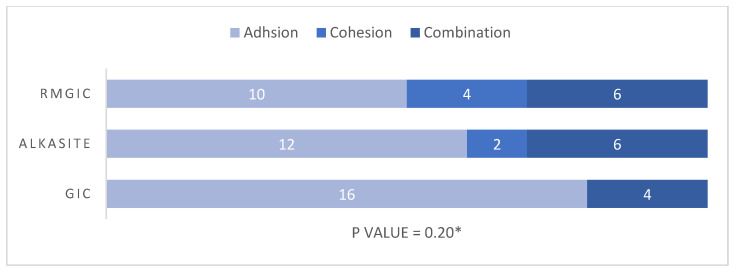
Delayed mode of failure. * Obtained using Fisher’s exact test.

**Figure 5:**
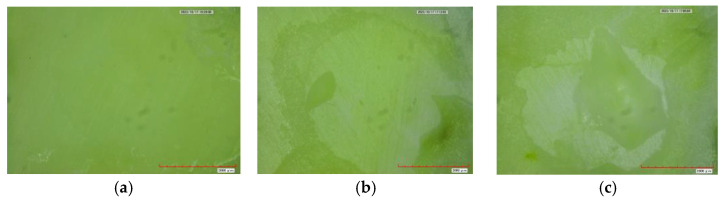
Photographs obtained using a digital microscope (50×) showing the (**a**) adhesion, (**b**) cohesion, and (**c**) combination modes of failure.

The immediate and delayed modes of failure of the study materials are presented in Figure 3 and Figure 4, respectively. Adhesion failure was observed to be the major mode of failure in all immediate tested groups, namely 70% in the RMGIC and GIC groups and 60% in the Alkasite group. The cohesion modes of failure were observed to be 15% in the RMGIC and Alkasite groups and 30% in the GIC group. Lastly, the combination type in immediate SBS was observed to be 15% in the RMGIC group, 25% in the Alkasite group, and 0% in the GIC group. However, no statistically significant difference was observed in the mode of failure between the study materials (*p* = 0.16). Similarly, in the delayed tested groups, the adhesion mode of failure was found to be the predominant type, with 50% in the RMGIC group, 60% in the Alkasite group, and 80% in the GIC group, followed by the combination type, with 30% in the RMGIC group, 30% in the Alkasite group, and 20% in the GIC group. Cohesion failure was observed the least, with 20% in the RMGIC group, 10% in the Alkasite group, and 0% in the GIC group. However, no statistically significant difference was found in the mode of failure between the delayed groups (*p* = 0.20). Photographs of the different types of failure under a microscope are shown in Figure 5. A clear dentin surface is shown in Figure 5a, which represents adhesion failure, where complete separation between the tooth structure and the material happened. A separation between materials molecules as result of cohesion failure is shown in Figure 5b, where residuals of the material can be noticed on dentin surface. Finally, shown Figure 5c shows a combination between adhesion and cohesion failure, where part of the dentin surface is cleared and some of the material is still adhered to the other part.

## 4. Discussion

The objective of this study was to assess the shear bond strength of Alkasite (Cention N) restorative material and resin-modified glass ionomer cement (RMGIC) in comparison to traditional glass ionomer cement (GIC). The results of this study reveal that there is no significant difference in the shear bond strength between Alkasite restorative material and RMGIC in both the immediate and delayed tested groups. Conventional GIC showed the lowest bond strength in both the immediate and delayed groups. The higher shear bond strength exhibited by Alkasite and RMGIC indicates the influence of their respective compositions. This could be due to the various glass fillers present in the powder of Alkasite [6]. Moreover, Alkasite restorative material, known for its high filler loading and resin-based matrix, promotes enhanced adhesion to the tooth structure. Similarly, RMGIC, which consists of a combination of glass ionomer and resin components, offers improved bond strength attributed to its resin content. These characteristics contribute to the increased bond strength observed when compared with traditional GIC [18].

Multiple in vitro studies compared Cention N restorative material with others using permanent teeth and observed similar findings. Kumari et al. [19] compared Cention N with conventional GIC (GC Fuji II) and Zirconomer using premolars and concluded that Alkasite showed the highest bond strength, while GIC showed the lowest bond strength. Moreover, Naz et al. [20] conducted an in vitro study comparing the bond strength of premolars using Cention N, Filtek (nano-hybrid composite), and GC Fuji IX (GIC). They found no significant difference between the Cention N and Filtek groups. Also, the Cention N group exhibited the highest mean value, and the Fuji IX group exhibited the lowest value.

Shear bond strength refers to the maximum stress that a material can bear before experiencing failure under shear loading conditions. This property is especially significant when studying the interfaces between two different materials. Understanding the shear strength helps in assessing the ability of materials to withstand forces that cause them to slide or deform along their planes of contact. By evaluating the shear strength, the stability and durability of material interfaces will be identified, which are critical for various applications and structural integrity [21]. The mean shear bond strength of RMGIC material is significantly higher than that of conventional glass ionomer material in both the immediate and delayed groups, which could be due to the superior wetting ability of hydroxyethyl methacrylate (HEMA) [22]. The final mean values of shear bond strength in dental restorative materials can be affected by several factors, including the condition of the mineralized dentin, the technique used for etching, the type of composite and adhesive materials used, the dentine depth, the moisture conditions during bonding, the curing mode, and the storage time after restoration [23]. A higher shear bond strength indicates a stronger bond between the material and the tooth structure [24]. The effectiveness of restorative materials relies on their ability to bond to the tooth structure and withstand different clinical stresses and shearing forces created by the interface of the tooth [24].

Artificial aging is common when testing materials’ in vitro shear and tensile bond strengths [25]. According to the literature, artificial aging may increase or decrease the physical properties of tested materials. This was observed by Scherer et al. [26] and Helvatjoglu et al. [27], who concluded that the physical properties of their tested materials were significantly decreased after artificial aging. However, artificial aging might increase some of the material’s mechanical properties, as observed by Prado et al. [28]. Moreover, many researchers concluded that artificial aging had no significant effect on the material’s mechanical properties, as observed in some studies, such as that by Cetin et al. [29], who found no significance between the mean of the mechanical load cycling group and control group. Benalcazar et al. and [30] Burger et al. [31] also did not find a significant difference between their tested groups, and this finding is applicable to this study, which also observed no significance.

Thermal curing of Cention N restorative material is recommended by the manufacturer as it facilitates a faster clinical procedure, improving efficiency for dental practitioners [6]. While thermal curing is not a mandatory step for either Cention N or GIC restorations, studies have shown that applying different temperatures during the curing process can significantly enhance the material’s mechanical properties [32,33]. Several studies have demonstrated that GIC restorative materials exhibit low thermal conductivity, ensuring that they do not irritate the pulp when applied to vital teeth, as observed by Gavic et al. [34] and van Duinen et al. [35]. In this study, both Cention N and GIC restorations were subjected to a 10 s curing period, anticipating an improvement in shear bond strength with this approach.

To assess the types of failure, a stereomicroscope and scanning electron microscope (SEM) were utilized in this investigation. In the present study and others [36,37], adhesion failure was observed to be the main mode of failure in both the immediate and delayed groups. Evaluating bond failure can provide insights into the bond’s nature between tooth molecules and the molecules of restorative material [38]. The disruption in connection between tooth and material molecules will result in adhesive failure [38], whereas cohesive failure occurs when the bond is disconnected between the molecules of the same material or tooth [38]. It is believed that the correlation of cohesive failure is directly proportional to the strength of the bond [38]. However, the actual performance of a restoration material in clinical settings differs significantly from what can be observed in controlled laboratory settings, since it is nearly impossible to fully replicate intraoral conditions and stress. Therefore, it is important to acknowledge that the findings of this in vitro study may not directly reflect the clinical performance of the material. Consequently, it is suggested that additional research be carried out to assess the material’s clinical performance in real-life conditions as the analysis of failure modes is valuable when interpreting results [39].

This study contributes to the existing literature by providing novel insights into the performance of Alkasite in primary teeth, particularly its SBS post-artificial aging, a topic previously unexplored. This study contributes new knowledge and insights to the field of dental materials and bonding techniques. On the other hand, the fact that this study was conducted in a laboratory setting is a limitation. While laboratory studies provide valuable insights and controlled conditions, they may not fully replicate the complex and dynamic nature of clinical environments. Therefore, the findings should be cautiously extrapolated to real-world clinical scenarios. It is important to consider that shear bond strength alone may not be the sole determinant of clinical success. Other factors, including the material’s mechanical properties, such as bending and tensile strength, esthetics, biocompatibility, and handling characteristics, should be considered when selecting a restorative material. Additionally, long-term clinical studies are warranted to evaluate the clinical performance and durability of Alkasite restorative material and RMGIC in comparison with GIC. Further research is required to comprehensively evaluate the performance and longevity of these materials. Clinical trials can provide a comprehensive understanding of the efficacy and safety of the Alkasite material in practical dental applications.

## 5. Conclusions

The following conclusions can be made: First, Alkasite restorative material showed a promising outcome; it can be used as an alternative to RMGIC and achieves a better result than GIC restorative material. Second, no statistically significant difference was found between immediate and delayed SBS. And third, the adhesive mode of failure is the most common among all materials in both the immediate and delayed subgroups.

## Figures and Tables

**Table 1:** Materials used in this study.

Material	Company	Chemical Composition	Manufacturer’s Instructions
Riva light cure, resin-modified glass ionomer cement—(RMGIC)	SDI Limited, Victoria, Australia	Compartment 1: 2-hydroxyethyl methacrylate (20–25%), polyacrylic acid (15–25%), dimethacrylate cross-linker (10–25%), and tartaric acid (1–5%). Compartment 2: Glass powder (95–100%) [8].	1. Apply Riva conditioner for 10 s; 2. Rinse with water, remove, and avoid dehydration; 3. Push plunger to activate capsule; 4. Mix for 10 sections; 5. Place capsule in applicator; 6. Light cure for 20 sections by using light-curing device (470 nm wavelength) [9].
Cention N (Alkasite)	Ivoclar Vivadent, Zurich, Switzerland	Liquid: Dimethacrylates, initiators, stabilizers, additives, and mint flavor. Powder: Calcium fluoro-silicate glass, barium glass, calcium barium aluminum fluoro-silicate glass, iso-fillers, ytterbium trifluoride, initiators, and pigments [10].	1. Shake powder bottle; 2. Apply one drop of liquid and one spoon of powder (1:1); 3. Mix; 4. Apply it in cavity; 5. Light cure [11].
Riva self-cure, conventional glass ionomer cement (CGIC)	SDI Limited, Victoria, Australia	Compartment 1: Polyacrylic acid (20–30%) and tartaric acid (10–15%). Compartment 2: Fluoro aluminosilicate glass (90–95%) and polyacrylic acid (5–10%) [8].	1. Apply Riva conditioner for 10 s; 2. Rinse with water, remove, and avoid dehydration; 3. Push plunger to activate capsule; 4. Mix for 10 sections; 5. Place capsule in applicator; 6. Wait for 6 min; 7. Light cure for 10 sections [10].

**Table 2:** The immediate shear bond strength of the tested materials.

Study Group	N	Mean in Mpa	SD	*p* Value *
RMGIC	20	10.64	1.74	<0.0001
Alkasite	20	10.84	1.96	
GIC	20	6.09	1.75	

* Obtained using one-way ANOVA.

**Table 3:** Between-group comparisons for immediate SBS.

Between-Group Comparison	Difference Between Means	95% Confidence Limits		*p*-Value *
Alkasite vs. RMGIC	0.20	−1.19	1.58	0.94
Alkasite vs. GIC	4.75	3.36	6.13	<0.0001
RMGIC vs. GIC	4.55	3.17	5.93	<0.0001

* Obtained using Tukey’s HSD post hoc test.

**Table 4:** The delayed shear bond strength of the tested materials.

Study Group	N	Mean in Mpa	SD	*p*-Value *
RMGIC	20	5.56	1.38	<0.0001
Alkasite	20	6.30	1.44	
GIC	20	3.29	0.61	

* Obtained using one-way ANOVA.

**Table 5:** Between-group comparisons for delayed SBS.

Between-Group Comparison	Difference Between Means	95% Confidence Limits		*p*-Value *
Alkasite vs. RMGIC	0.75	−0.18	1.65	0.14
Alkasite vs. GIC	3.01	2.09	3.93	<0.0001
RMGIC vs. GIC	2.27	1.36	3.19	<0.0001

* Obtained using Tukey’s HSD post hoc test.

**Table 6:** The effect of aging on the shear bond strength in different study groups.

Study Group	Immediate Mean SBS (Mean ± SD)	Delayed Mean SBS (Mean ± SD)	*p* Value
RMGIC	10.64 ± 1.74	5.56 ± 1.38	0.91
Alkasite	10.84 ± 1.96	6.30 ± 1.44	0.46
GIC	6.09 ±1.75	3.298 ± 0.61	0.61

## Data Availability

The data presented in this study are available on request from the corresponding author due to ethical considerations.
